# Relationship between Potential Sperm Factors Involved in Oocyte Activation and Sperm DNA Fragmentation with Intra-Cytoplasmic Sperm Injection Clinical Outcomes

**DOI:** 10.22074/cellj.2016.4725

**Published:** 2016-09-26

**Authors:** Marziyeh Tavalaee, Abbas Kiani-Esfahani, Mohammad Hossein Nasr-Esfahani

**Affiliations:** 1 Department of Reproductive Biotechnology, Reproductive Biomedicine Research Center, Royan Institute for Biotechnology, ACECR, Isfahan, Iran; 2 Isfahan Fertility and Infertility Center, Isfahan, Iran

**Keywords:** Intra-Cytoplasmic Sperm Injection, Phospholipase Cζ, Fertilization, DNA Fragmentation

## Abstract

**Objective:**

The present study aimed to simultaneously evaluate the association between expression of three potential factors [post-acrosomal sheath WW domain-binding protein (PAWP), phospholipase Cζ (PLCζ), and truncated form of the kit receptor (TR-KIT)] as candidates of oocyte activation with fertilization rate and early embryonic development.

**Materials and Methods:**

In this experimental study, semen samples were collected from 35 intra-cytoplasmic sperm injection (ICSI) candidates and analyzed according to World Health Organization criteria (2010). Each sample was divided into two parts. The first part was processed for insemination by density-gradient centrifugation (DGC) and the second part was prepared for assessment of sperm morphology (Papanicolaou staining), DNA fragmentation [transferase dUTP nick end labeling (TUNEL)], and three Sperm-borne oocyte-activating factor (s) (SOAFs)-PLCζ, PAWP, and TR-KIT.

**Results:**

Significant positive correlations existed between the percentages of PLCζ, PAWP, and TR-KIT with fertilization rate. In addition, significant negative correlations existed between the percentage of DNA fragmentation with the percentages of PLCζ and PAWP. We did not find a relationship between percentages of PLCζ, PAWP, and TR-KIT with embryo quality and pregnancy rate (P>0.05). There was a significant negative correlation between percentage of DNA fragmentation with fertilization and embryo quality.

**Conclusion:**

Oocyte activation was associated with the studied sperm factors (PAWP, PLCζ, and TR-KIT). These factors might hold the potential to be considered as diagnostic factors in the assessment of semen samples to evaluate their potential to induce oocyte activation. In addition, we observed a significant association between DNA fragmentation with fertilization, as well as embryo quality and expression of PAWP and PLCζ, which indicated that men with high degrees of DNA fragmentation might require artificial oocyte activation. Whether such action should take place, and its cost and benefits should be evaluated in the future.

## Introduction

Perinuclear theca (PT) is a cytoskeletal coat over the sperm nucleus. Its biogenesis is strongly associated with acrosomal biogenesis. This coat contains sperm-borne oocyte-activating factor(s) (SOAFs)that is responsible for oocyte activation. During fertilization, when the sperm membrane fuses with the oolemma, PT is removed from the sperm head and SAOA factor(s) is released into the oocyte to induce oocyte activation (1). Several factors are introduced as potential candidate sperm factors involved in oocyte activation, such as phospholipase Cζ (PLCζ), a 33 kDa protein; truncated form of the kit receptor (TR-KIT); and post-acrosomal sheath WW domain-binding protein (PAWP) (2-6). These proteins are localized in the sperm head and mostly in the PT or post-acrosomal region. However there are contradictive reports between studies regarding the localization pattern of these factors in mouse, bull, and human sperm (7, 8).

Microinjection of recombinant bovine PAWP into the MII-arrested bovine, porcine, and Xenopus oocytes led to oocyte activation and pronuclear formation (9). Aarabi et al. (4) demonstrated that microinjection of recombinant human PAWP protein or complementary RNA (cRNA) into oocytes could induce Ca2+ oscillations in human and mouse oocytes. This group showed significant positive correlations between PAWP with fertilization rate and embryonic quality following intra-cytoplasmic sperm injection (ICSI) in humans (10). In addition, Kennedy (11) et al. have shown that strong correlations exist between PAWP with bovine sperm quality and fertility.

Another possible sperm factor involved in oocyte activation at fertilization is TR-KIT. It has been shown that microinjection of recombinant TR-KIT into mouse oocytes led to meiotic resumption, formation of pronuclei, and development up to the morula stage (3). In this regard, we did not find any study that evaluated the role of TR-KIT in fertilization and early embryonic development in a human model. Only Muciaccia et al. (2) showed that this protein localized in the equatorial region of human spermatozoa and a negative correlation existed between sperm DNA fragmentation and the TR-KIT protein.

Another candidate sperm factor responsible for oocyte activation is PLCζ. Microinjection of PLCζ RNA and PLCζ protein into mouse oocytes have also resulted in Ca2+ oscillations and early embryonic development (12). Our group previously showed significantly lower relative expression of PLCζ mRNA in infertile men with previously low or failed fertilization and those with globozoospermia compared to fertile men. There was a significant correlation observed between fertilization rate and relative expression of PLCζ in infertile men considered as candidates for ICSI. Therefore, we concluded that “relative expression of PLCζ might provide a useful marker for the ability of sperm to induce oocyte activation after ICSI” (13). Recently, Yelumalai et al. (14) observed significant positive correlations between localization patterns and percentage of sperm PLCζ with fertilization rate in infertile men who were candidates for ICSI but not candidates for *in vitro* fertilization (IVF) according to immunocytochemical analyses which emphasized the essential role of PLCζ in oocyte activation.

A literature search revealed the presence of a considerable number of clinical studies related to these factors in infertile men. The majority of these studies assessed a limited number of cases and the main focus of these studies was on one of the factors. The present study, for the first time, simultaneously evaluated the three main (PAWP, PLCζ, and TR-KIT) potential candidates involved in oocyte activation which subsequently have led to fertilization and early embryonic development. The flow cytometry assessment has been performed on semen samples from infertile individuals that referred for ICSI. Numerous studies have shown that sperm DNA fragmentation is an important cause of male infertility (15). Therefore, we have simultaneously assessed sperm DNA fragmentation and expression of these three sperm factors on the remaining semen samples of individuals that referred for ICSI with the intent to assess the relationship between these factors.

## Materials and Methods

### Semen sample collection and preparation

The Institutional Review Board of Royan Institute approved this study. Semen samples were obtained by masturbation after 3-5 days of ejaculatory abstinence into a sterile plastic container by 35 ICSI candidates who referred to Isfahan Fertility and Infertility Center on the day of oocyte retrieval. Informed consent forms were signed by all patients. The samples were liquefied for at least 20 minutes prior to routine semen analysis. Semen parameters were assessed according to World Health Organization Guidelines (16).

Each sample was separated into two portions. The first part was processed for insemination by densitygradient centrifugation (DGC) (17). The second part was prepared for research aims using a simple wash in phosphate-buffered saline (PBS) buffer. After removal of seminal plasma, the pellet was utilized for the assessment of sperm morphology (Papanicolaou staining), DNA fragmentation [transferase dUTP nick end labeling (TUNEL)], and three SOAFs-PLCζ, PAWP, and TR-KIT. All chemicals were obtained from Merck (Germany) unless otherwise stated.

### Intra-cytoplasmic sperm injection procedure and embryo scoring

All the media for the ICSI procedure were purchased from Vitrolife (G3 Series Plus, Sweden). Ovarian stimulation, ICSI procedure, fertilization rate, embryo scoring, and pregnancy rate were carried out according to Kheirollahi-Kouhestani et al. (17). We excluded couples with female-factor infertility or females with <4 mature metaphase II oocytes for ICSI.

### Flow cytometry analyses of phospholipase Cζ, post-acrosomal sheath WW domain-binding protein and truncated form of the kit receptor

Briefly, semen samples were washed in PBS and fixed in cold acetone for assessment of TR-KIT and in 4% paraformaldehyde for evaluation of PAWP and PLCζ. Then, sperm pellets were washed twice with PBS for 5 minutes at 3000 rpm. For PAWP and PLCζ, we treated the pellets with 0.5% Triton X-100 for 30 minutes; after washing with PBS, the pellets were incubated with 3% bovine serum albumin (BSA)/ PBS for 1 hour to block non-specific binding sites. For TR-KIT, sperm pellets were incubated with 5% BSA+10% normal goat serum (NGS) for 2 hours to block non-specific binding sites. Then, the affinitypurified anti-human primary antibodies [PLCζ (Covalab/ France), PAWP (Abcam, Cambridge) and TRKIT (Santa Cruz, Europe)] in PBS that contained 1% BSA were applied overnight at 4˚C. After washing with PBS, samples were incubated with goat antirabbit IgG secondary antibody FITC conjugated for 1 hour at 37˚C. Ultimately, samples were washed with PBS and propidium iodide (1 μg/ml) was used as a counterstain. The percentage of PAWP, PLCζ and TRKIT in the propidium iodide positive sperm population were assessed by a FACSCalibur flow cytometer (Becton Dickinson, San Jose, CA, USA) using an argon laser with an excitation wave length of 488 nm. A minimum of 10000 sperm were examined for each assay and analyzed with BD CellQuest Pro software. Assessments of the three main SOAFs (PLCζ, PAWP and TR-KIT) were performed according to modified protocols by Aarabi et al. (10), Grasa et al. (18), and Muciaccia et al. (2), respectively.

### Assessment of DNA fragmentation by the transferase dUTP nick end labeling assay

Sperm DNA fragmentation was assessed using a detection kit (Apoptosis Detection System Fluorescein, Promega, Germany). Semen samples were washed twice in PBS, pH=7.4. A droplet of this sperm suspension was smeared onto slides, air-dried and fixed by 4% paraformaldehyde for 25 minutes. Then, slides were washed in PBS and treated with 0.2% Triton X-100 for 5 minutes. After washing in PBS, the procedure was carried out according to the manufacturer’s instructions. For each sample, at least 200 sperm were randomly assessed using an Olympus fluorescent microscope (BX51, Japan) with the appropriate filters (460-470 nm) at ×100 magnification. The percentage of green fluorescing sperm (TUNEL positive) was defined as sperm with DNA fragmentation (17).

### Statistical analysis

The Statistical Package for Social Sciences (SPSS) version 16 (SPSS Science, Chicago, IL, USA) and Microsoft Excel 2010 spread sheet were used for data entry and analysis. Two-tailed Pearson correlation test was used to assess the correlations between parameters. P<0.05 was considered statistically significant. The results in the text and figures are presented as mean ± SEM.

## Results

The studied population consisted of 35 infertile individual candidates for ICSI. Table 1 shows the descriptive information of the semen parameters.

We assessed PLCζ, PAWP, and TR-KIT as potential candidates of SOAFs by flow cytometry and evaluated the correlations between these factors and conventional sperm parameters. As shown in Table 2, the percentage of PAWP positive sperm significantly correlated with sperm concentration (r=0.464, P=0.007), percentage of motility (r=0.402, P=0.02), and percentage of abnormal morphology (r=-0.508, P=0.003). In addition, we observed significant correlation between the percentage of PLCζ positive sperm and sperm concentration (r = 0.46, P=0.005). No statistically significant correlations existed between the percentage of PLCζ positive sperm with percentage of motility or percentage of abnormal morphology. We observed no significant correlations between the percentage of TR-KIT positive sperm and semen parameters.

**Fig.1:**
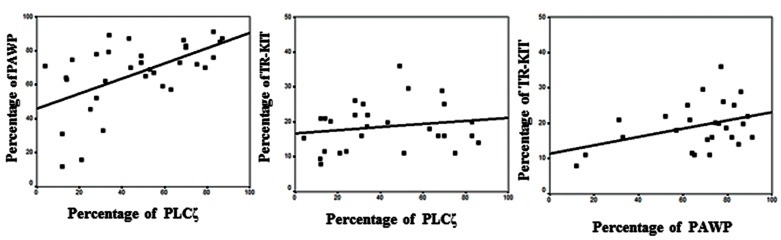
Correlations between percentages of phospholipase Cζ (PLCζ) and post-acrosomal sheath WW domain-binding protein (PAWP, r=0.56, P=0.001), PLCζ and truncated form of the kit receptor (TR-KIT, r=0.4, P=0.16) and PAWP and TR-KIT (r=0.38, P=0.05).

We also observed significant correlations between percentage of PAWP positive sperm with PLCζ (r=0.56, P=0.001) and TR-KIT (r=0.38, P=0.05) as depicted in Figure 1.

**Fig.2:**
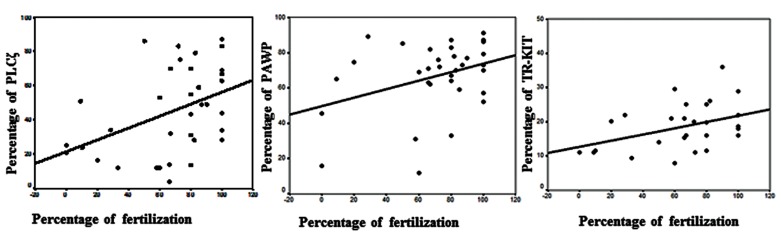
Correlations between percentages of phospholipase C ζ (PLCζ, r=0.42, P=0.01), post-acrosomal sheath WW domain-binding protein (PAWP, r=0.35, P=0.04), and truncated form of the kit receptor (TR-KIT, r=0.4, P=0.03) with fertilization rate.

**Fig.3:**
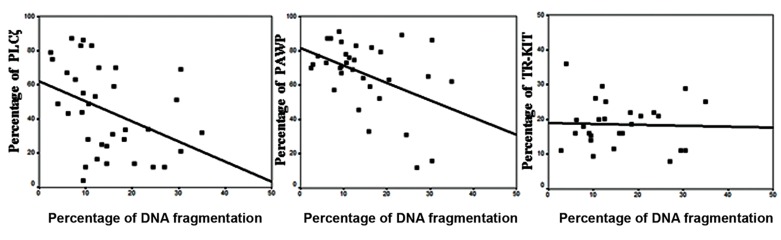
Correlations between percentage of phospholipase C ζ (PLCζ, r=-0.4, P=0.02), post-acrosomal sheath WW domain-binding protein (PAWP, r=-0.44, P=0.01), and truncated form of the kit receptor (TR-KIT, r=-0.36, P=0.85) with DNA fragmentation.

**Fig.4:**
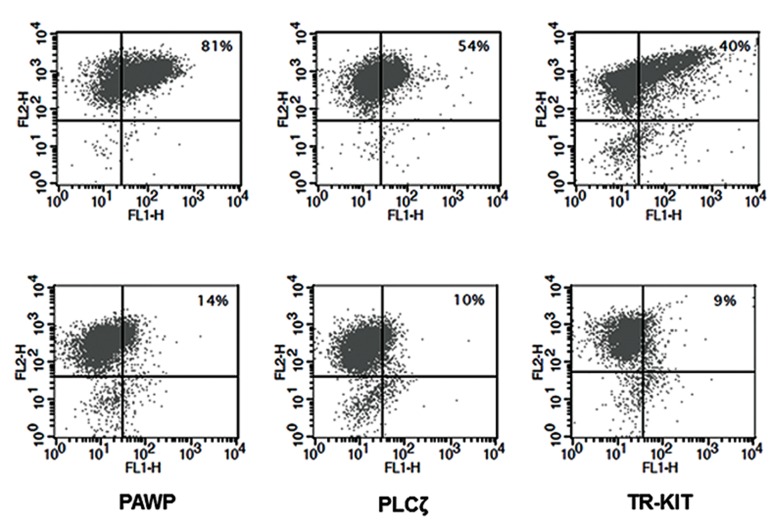
Comparison of percentages of phospholipase Cζ (PLCζ), post-acrosomal sheath WW domain-binding protein (PAWP), and truncated form of the kit receptor (TR-KIT) in two samples with A. High fertilization (70%) and B. Low fertilization (15%) by flow cytometry.

In this study, we analyzed the correlations between the percentage of PLCζ, PAWP, and TR-KIT with fertilization rate (Fig .2) and DNA fragmentation (Fig .3). There were statistically significant positive correlations between the percentage of PLCζ (r=0.424, P=0.01), PAWP (r=0.356, P=0.04), and TRKIT (r=0.404, P=0.03) with fertilization rate. In addition, there were significantly negative correlations between the percentage of DNA fragmentation with percentage of PLCζ (r=-0.391, P=0.02) and PAWP (r=-0.441, P=0.01). While no association was observed between percentage of TR-KIT positive sperm (r=-0.036, P=0.85) with DNA fragmentation. Figure 4 compares the percentage of PLCζ, PAWP, and TRKIT in two samples with high fertilization (70%) and low fertilization (15%) by flow cytometery.

We did not find a relationship between percentage of PLCζ, PAWP, and TR-KIT with embryo quality (P>0.05). There was a significantly negative correlation observed between percentage of DNA fragmentation with embryo quality (r=-0.386, P=0.047). In addition, we compared the percentage of PLCζ, PAWP, and TR-KIT between individuals who achieved clinical pregnancy to those that did not achieve pregnancy. There was no difference observed between the two groups. From 35 patients, 16 patients had embryo transfers, 7 patients were cancelled, and 15 patients had no embryo transfers. Clinical pregnancy was confirmed by ultrasound and out of 16 patients, 6 patients achieved pregnancy, which resulted in a pregnancy rate of 37.5%.

**Table 1:** Descriptive analysis of semen parameters in 35 infertile male candidates for intra-cytoplasmic sperm injection (ICSI)


Parameters	Minimum	Maximum	Mean ± SE

Sperm concentration (10^6^/ml)	6.00	200.00	72.65 ± 5.57
Sperm motility (%)	5.50	75.00	57.30 ± 2.61
Abnormal morphology (%)	94.00	100.00	95.97 ± 0.27
Volume (ml)	1.20	9.30	4.78 ± 0.31
Male age (Y)	23.00	53.00	38.00 ± 1.36
Female age (Y)	21.00	46.00	32.87 ± 1.03


**Table 2:** Correlations between semen parameters and percentage of phospholipase Cζ (PLCζ), post-acrosomal sheath WW domain-binding protein (PAWP), and truncated form of the kit receptor (TR-KIT) positive sperm.


Parameters	PLCζ		PAWP	TR-KIT

Sperm concentration (10^6^/ml)	R	0.46	0.464	0.249
	P	0.005	0.007	0.201
Sperm motility (%)	R	0.29	0.402	0.02
	P	0.08	0.02	0.89
Abnormal morphology (%)	R	-0.13	-0.508	-0.11
	P	0.45	0.003	0.57


R; Pearson correlation and P; Significance (2-tailed).

## Discussion

Despite the injection of a sperm into the oocyte during ICSI, leading to an average fertilization rate of approximately 70-80% (19), failure of fertilization still occurs in a small percentage of couples. The rate of total failed fertilization (TFF) comprises 1-3% of ICSI cycles (20) and is mostly due to oocyte activation failure (21). There are strong indications that activation of oocytes after ICSI is related to the release of SOAFs from the PT region of the sperm head such as PLCζ, PAWP, and TR-KIT into the oocyte (22, 23). A close correlation exists between the acrosome and PT biogenesis (1). In light of this consideration, several studies have demonstrated that infertile men with globozoospermia commonly have defects in acrosome biogenesis, chromatin remodeling, cytoskeleton and oocyte activation (24-26). Low expressions of PLCζ at the RNA and protein levels were reported in these individuals due to absence or deficiency in acrosome and PAS-PT biogenesis (27-29). Therefore, ICSI followed by artificial oocyte activation has been shown to improve fertilization and pregnancy outcomes in these types of infertile couples (29). However they are not the only couples that face low fertilization or total fertilization failure. It has been shown that couples with high degree of sperm anomalies may also face high degree of fertilization failure. In addition, there are reports on couples with normal semen parameters that have TFF (30). Therefore, assessment of sperm factors involved in oocyte activation as a potential predictor of successful fertilization may help with selection of candidate couples for artificial oocyte activation. This study has aimed to simultaneously evaluate the association between expressions of three potential factors (PAWP, PLCζ, and TR-KIT) candidates of oocyte activation with fertilization rate and early embryonic development.

In this study, we observed significant correlations between fertilization rate with the percentages of PAWP, PLCζ, and TR-KIT positive sperm in infertile male candidates for ICSI. These data suggested that couples who presented with a low degree expression of these protein were more likely to face low fertilization or total fertilization failure post-ICSI. We previously showed a significant correlation between fertilization rate and expression of PLCζ at the mRNA level in infertile men (13). This result agreed with a recent study which showed a significant positive correlation between ICSI fertilization rates, but not IVF, with percentage of sperm that exhibited PLCζ at the protein level (31).

Recently, a study published by Aarabi et al. (10) reported significant correlations between expression of PAWP with fertilization rate and embryo quality. These authors stated that "low levels of PAWP could possibly lead to shortened/impaired calcium oscillations and hence cause the arrest of embryonic development". Unlike fertilization rate, we did not observe any association between the sperm factors involved in oocyte activation with embryo quality. Therefore, based on our results and previous studies PAWP, like PLCζ, might have significant diagnostic and prognostic value for sperm quality, as well as a potential candidate for the mammalian 'sperm factor'. TR-KIT also showed a significant correlation with fertilization. According to the literature, studies on mice revealed that TR-KIT was associated with oocyte activation, however reports on humans were very scarce (32). Our results clearly showed that sperm factors assessed in this study played an important role in activation of the oocyte, pronucleus formation, and fertilization but not with embryonic development. In addition, we did not observe any difference between men in expression of these sperm factors between the pregnant and non-pregnant groups. This result supported findings by Aarabi et al. (10) in which no correlation existed between pregnancy rate and PAWP.

In this study we observed significant correlations between percentage of sperm DNA fragmentation with fertilization and embryo quality after ICSI. Fertility failure in some couples might be due to fragmented DNA in sperm. Spermatozoa with fragmented DNA can be alive, mature, morphologically normal, and have the capability to fertilize an oocyte (33, 34). However, numerous studies have shown significant negative correlations between elevated sperm DNA fragmentation with embryo cleavage, implantation rate, miscarriage rates, and pregnancy loss after IVF and ICSI (35-37). Fernandez-Gonzalez et al. (38) demonstrated that injection of sperm with high DNA fragmentation could lead to abnormal behavior, malformations, and signs of premature aging in adult mice born and cancer risk in future generations. Our results also showed that low fertilization and low level of embryo quality were possible consequences of injected sperm with damaged DNA into the oocytes. In this regard, Wdowiak et al. (33) reported that high levels of sperm DNA fragmentation could affect embryo morphokinetic parameters. Embryos from infertile couples with low levels of damaged DNA in sperm who achieved pregnancy, grew faster compared to infertile couples with high levels of sperm DNA damaged who did not achieve pregnancy. However, there are indications that the oocyte is able to repair sperm DNA damage, but it is strongly related to extent of sperm DNA fragmentation, age, and quality of the oocyte (39, 40). In addition, we have observed significant correlations between percentages of sperm DNA fragmentation with percentages of PAWP and PLCζ. Reactive oxygen species (ROS) is considered one of the main factors involved in induction of DNA fragmentation (41). Recently, a report by Park et al. (42) showed that oxidative stress had detrimental effects on PLCζ expression as a SAOA factor which was consistent with our observation. Possibly, due to DNA damage, the expression of these factors might reduce RNA expression which resulted in lower translation and lower expression of these proteins. This possibility could also explain the significant correlations observed between the three semen parameters with the percentage of PAWP positive sperm and between sperm concentrations with the percentage of PLCζ positive sperm.

## Conclusion

The results of this study clearly revealed that the main cause of failed fertilization was failure in oocyte activation due to the low percentage of sperm factors involved in oocyte activation (PAWP, PLCζ and TR-KIT). These factors might hold the potential to be considered as diagnostic factors in assessment of semen samples in order to evaluate their potential to induce oocyte activation. In addition we observed a significant association between DNA fragmentation with fertilization, embryo quality, and expressions of PAWP and PLCζ. These results indicated that men with high degrees of DNA fragmentation might require oocyte activation. Whether such action should take place, and its cost and benefits remain to be evaluated in the future.
